# When algorithms testify: artificial intelligence-driven DNA analysis, evidentiary standards, and criminal justice reform

**DOI:** 10.3325/cmj.2026.67.203

**Published:** 2026-06

**Authors:** Dragan Primorac, Damir Primorac, Andrej Bozhinovski

**Affiliations:** 1 St. Catherine Specialty Hospital, Zabok/Zagreb, Croatia; 2 Eberly College of Science, The Pennsylvania State University, University Park, PA, USA; 3 University of Split School of Medicine, Split, Croatia; 4 University of Osijek School of Medicine, Osijek, Croatia; 5 University Department for Forensic Sciences, University of Split, Split, Croatia; 6 University of Mostar, Law School, Mostar, Bosnia and Herzegovina; 7 Department of Criminal Law, Faculty of Law, University of Zagreb, Zagreb, Croatia

## Abstract

Forensic DNA analysis has already influenced criminal justice, and serves as a powerful tool for both conviction and exoneration. Despite its scientific foundations and wide application, DNA evidence is vulnerable to interpretive errors, methodological limitations, and cognitive bias, as demonstrated by numerous wrongful convictions identified through the Innocence Project. Recent artificial intelligence (AI) methods, especially probabilistic genotyping, are used to support the interpretation of complex DNA samples, including mixed, low-template, and degraded profiles. However, the repeated utilization of AI-driven forensic analysis can lead to legal and ethical concerns, including procedural challenges in terms of its usability as direct evidence in the procedure. This article examines the implications of AI-based DNA interpretation for criminal justice, with particular attention to evidentiary reliability, due process, institutional accountability, and emerging policy responses in the US and Europe. It draws on parallels with clinical genomics and documented forensic applications of AI, and argues that AI can enhance forensic accuracy and fairness only if integrated within transparent, validated, and ethically governed frameworks that respect fundamental legal protections.

DNA technology marked a paradigm shift in criminal proceedings. As technology improved and DNA databanks with forensic materials were developed, more and more innocent individuals gained the benefit of this so-called miracle science (1). By the 1990s, the ability to match DNA profiles propelled the innocence work, and the first DNA-centered Innocence Project was established within the Cardozo School of Law at Yeshiva University in New York City (2). A similar project was carried out in Europe, showing that even European criminal justice systems, with all the necessary fair trial guarantees, are capable of generating wrongful convictions (2). Just as DNA once transformed the way criminal justice actors use scientific methods and technologies, today we witness another technological breakthrough. Artificial intelligence (AI) promises to reshape how complex forensic DNA evidence is used and interpreted in the criminal proceedings, especially with the new enhanced probabilistic genotyping. AI systems and statistical models are able to assess complex DNA samples such as mixtures of DNA traces, or low-template materials, making probabilistic ratios much more precise. AI cannot replace the human factor, but its growing use in forensic science can improve the handling of complex DNA data. However, every new technology needs to be properly integrated into existing procedural standards. This raises the core questions that this article aims to address: What can be considered as an AI-driven DNA analysis?; What are the evidentiary standards for the admittance of new AI-analyzed scientific evidence in the criminal proceedings and can AI methods be evaluated as a new scientific method?; If so, to what extent and how is this relevant to innocence work?. In addition, the article explores the relevant legal and policy framework and jurisprudential implications of using such systems in the US and Europe, as well as what this means for the ethical use of AI in probabilistic genotyping, and overall future of the criminal justice reform.

DNA profiling is often treated by courts and fact-finders as highly persuasive scientific evidence because it appears objective, quantifiable, and rooted in molecular biology. However, post-conviction investigations have shown that DNA evidence can be misinterpreted, overstated, or presented without adequate acknowledgment of uncertainty. AI is increasingly being applied to forensic DNA analysis to address longstanding challenges in the interpretation of complex biological data. Similar computational approaches have already transformed clinical genomics, rare-disease diagnostics, and oncology (3). The transition of AI tools from medicine to forensic practice represents a critical juncture that demands careful assessment of scientific reliability, legal safeguards, and ethical responsibility. Having this in mind, this paper examines the use of AI-assisted methods in forensic DNA interpretation, focusing on probabilistic genotyping, an emerging evidentiary and procedural phenomenon linking forensic science and criminal justice. The central argument is that AI-assisted DNA analysis can improve the interpretation of complex biological evidence and strengthen post-conviction review only if its use is embedded within transparent, scientifically validated, explainable, and procedurally accountable legal frameworks that preserve due process and equality of arms.

This article employed interdisciplinary doctrinal and normative methodology. It compared US and European approaches to AI-assisted evidentiary reliability, admissibility, and evaluation of such evidence. Furthermore, it assessed the treatment of such evidence in the courtroom and implications for defense rights of defendants by taking insights from forensic genetics, clinical genomics, and legal and policy framework of AI governance. The doctrinal research component focuses on academic sources, field reports, and jurisprudence relevant to the use of AI-assisted DNA analysis. The normative component evaluates whether existing procedural legal safeguards are sufficient to ensure adherence to the defense rights and fair trial. To support and illustrate the legal argument, the article also draws on selected jurisprudence from US and European jurisdictions, involving probabilistic genotyping, treatment of DNA evidence, and post-conviction DNA review.

This article is designed to answer to whether AI-assisted DNA analysis, particularly probabilistic genotyping, can be used in criminal proceedings in a way that improves forensic accuracy without undermining due process and equality of arms. It first explains the relationship between DNA evidence and wrongful convictions, then examines the emergence and operation of AI-assisted forensic DNA interpretation and whether clinical genomics can be used as a model for responsible AI implementation. It further analyzes the policy approaches to AI in legal systems, ethical responsibilities, and future trends. As an axiological component, the article analyzes its evidentiary, procedural, and policy implications within the US and European jurisdictions. The conclusion explains why AI-assisted DNA analysis, especially probabilistic phenotyping, should be admitted into court, and how it can benefit innocence work.

## The emergence of AI in forensic DNA analysis

DNA evidence has a distinctive position in criminal justice because it may lead to establishing both guilt and innocence (4). This is why, even in continental European systems, DNA evidence is often regarded as particularly powerful and outcome-determinative, capable of justifying the reopening or overturning of a final judgment (5). By contrast, other types of evidence are more often treated as merely a reinterpretation of the material already presented. The role of AI in forensics is called probabilistic genotyping, which is software-assisted statistical modeling to interpret mixed, low-template, or complex DNA samples gathered from the crime scene, and estimate the strength of the proposed contributor’s connection to the sample (6).

Although there are several definitions of AI, all of them agree that the term *AI* should be understood as broadly as a group of computational methods capable of processing data and producing outputs that may support human decision-making. Automated decision-making (ADM) systems within the AI context are relevant because they describe a wider category of technologies that use data inputs to generate outputs capable of shaping, supporting, or in some cases, influencing decisions made by humans. The Council of Europe (CoE) has described AI “as an umbrella term to refer generally to a set of sciences, theories, and techniques dedicated to improving the ability of machines to do things requiring intelligence (7). An AI system is a machine-based system that makes recommendations, predictions or decisions for a given set of objectives. It does so by: (i) utilizing machine and/or human-based inputs to perceive real and/or virtual environments; (ii) abstracting such perceptions into models manually or automatically; and (iii) deriving outcomes from these models, whether by human or automated means, in the form of recommendations, predictions or decisions” (8). Furthermore, “the EU High-Level Expert Group on AI describes AI as software and hardware systems designed by humans that, given a complex goal, act in the physical or digital dimension by perceiving their environment through data acquisition, interpreting the collected structured or unstructured data, reasoning on the knowledge, or processing the information, derived from this data and deciding the best action(s) to take to achieve the given goal (9). Therefore, AI systems can either use symbolic rules or learn a numeric model, and they can also adapt their behavior by analyzing how the environment is affected by their previous actions*.”* According to Garret, there are two models of AI use: the black-box model and the glass-box model (10). The CoE recommendations describe a black box as a system whose internal reasoning is not meaningfully understandable to judges, lawyers, defendants, or sometimes even its own developers, which can hide bias, inaccuracies, or other defects (7,11). In forensic DNA, this concern appears with probabilistic genotyping software, where courts may receive a likelihood ratio without full insight into the assumptions, settings, or model logic behind it (12). On the other hand, the glass box effect refers to environments where all processes are transparent and understandable. In criminal justice, this matters because the court can better test the basis of a result rather than treating the algorithm as an authority that simply must be trusted.

So, how is AI used in forensic examinations? Garret explains that the task for forensic practice is to determine whether biological material recovered from a crime scene can be associated with a particular individual. Where investigators lack a suspect, forensic material may be compared with existing databases, including DNA profiles, fingerprints, or any other trace evidence (10). Furthermore, in the context of DNA databases, a numerical DNA profile containing the person’s genetic markers may be entered into a DNA profile database (10). These tests rely on genetic markers that are highly variable within the population, making them useful for linking biological evidence to specific individuals. AI has entered the courtroom primarily in the analysis of complex DNA mixtures, including samples involving several contributors or an unknown number of contributors. In such cases, algorithms interpret DNA test results and assess whether a suspect may or may not have contributed to the crime scene sample. The scientific community has found probabilistic genotyping promising, but not yet well validated outside certain well-defined ranges (10). The results gathered through these DNA mixtures have been introduced in court as expert evidence (10). However, when asked to be transparent on how these systems have reached these results, experts reiterated that the phenotyping software was proprietary, and cannot be shared for the purposes of evaluation (10). This is the definition of black-box AI, and judicial practices permit their use in criminal proceedings based on the admissibility standards for scientific evidence. In the glass-box AI model, on the other hand, the processes are widely explained and open to the parties of the procedure. They described the path of deduction and reasoning, and concluded the parameters how the decision was made.

When it comes to probabilistic genotyping, the Federal Judicial Center explains that the software starts with an evidence sample containing DNA and analyzes which combinations of contributors could plausibly explain the pattern (12). The gathered results are then compared against statistical modeling to test possible genotype combinations, and compare explanations for the data that were subject to observation. The most used software packages in this sense are STRmix and TrueAllele. The output is the likelihood ratio, comparing two propositions: the degree of probability of observing the DNA profile if the subject contributed to the mixture and the degree of probability of observing the DNA profile if the subject did not contribute, and instead a third-party contributor did. The software provides a probabilistic rather than a certain value, by stating that this DNA pattern is represented more than the other patterns. The expert then determines how this will be presented to court. The process is not fully automatic, as the forensic analyst oversees the important decisions. Also, the laboratories using this software are not selected randomly. There is a process of validation and standards. The Scientific Working Group on DNA Analysis Methods has published specific Guidelines for the Use of Probabilistic Genotyping with Autosomal STR Typing Results, as well as the Guidelines for the Validation of Probabilistic Genotyping Systems. This further reflects the field’s expectation that laboratories must validate the software before using it (13). Probabilistic genotyping has been adopted by more than 100 state and federal forensic laboratories and is present in other European jurisdictions as well. Its use is particularly common in sexual assault cases, firearm investigations, and violent crimes, where DNA mixtures and low-template samples are frequent (12,13).

## Clinical genomics as a model for responsible AI implementation

Clinical genomics can be used as a model for responsible implementation of AI as an assistant tool in DNA analysis. It provides a valuable framework for understanding how AI can be responsibly integrated into high-stakes decision-making. In medical practice, AI assists with variant interpretation, genotype-phenotype correlation, and prioritization of clinically relevant findings. Clinical genomics insists on strict standards and normalizes the idea that the AI system should operate inside a controlled institutional framework. It can be a model for the responsible implementation of AI because it already uses AI software as a decision support mechanism. AI is used in this segment to identify patterns in complex genomics data sets and to support clinical diagnostics, management therapeutics, and clinical support (14). In order to do this, clinical genomics had built oversight mechanisms that control the validation practices, and the governance of the algorithmic decision-maker according to the National Human Genome Research Institute (15). To this end, the World Health Organization further noted that AI in health should be accompanied by ethics, human rights, and accountability, which would include public oversight and transparency (16). Along the same lines, the National Institute of Standards and Technology (NIST) stipulates that genomic data environments are not ordinary data systems and that they should be classified as highly sensitive. For this reason, NIST frames trust in genomic systems around the need to manage risks across the entire genomic data life cycle, with particular attention to privacy protection and governance controls. Taking these experiences, clinical genomics can be considered as an example of AI implementation in a high-risk domain. This can be relevant in providing expert reporting, where the expertise should be made in a controlled framework for validation, and human oversight, as well as transparency (17). Forensic laboratories should follow NIST guidelines when relying on probabilistic genotyping and any other AI assistive tools. They should also have established clear validation procedures to determine which methods are scientifically reliable and acceptable, and in which circumstances they should be used. This includes identifying the conditions in which interpretation may be compromised by sample complexity, low-template DNA, mixed profiles, contextual bias, or human error (17). However, NIST is encouraging the scientific interpretation in criminal proceedings to be transparent, reliable, reviewable, and institutionally controlled (18).

## DNA evidence and wrongful convictions: transformative impact of AI-assisted DNA analysis in innocence litigation

Since the introduction of forensic DNA testing, hundreds of wrongfully convicted individuals worldwide have been exonerated (1). Brooks argues that crime-scene DNA is often contaminated, mixed, or present in trace amounts, which pushes traditional interpretation methods beyond their reliable limits. At the same time, the institutional context of criminal proceedings, marked by adversarial incentives, asymmetries in access to expertise, and deference to forensic authority, can transform scientific uncertainty into legally decisive claims. This presents a systemic risk to the integrity of fact-finding process of the court. To this end, post-conviction DNA testing has therefore become one of the most effective mechanisms for correcting wrongful convictions (19). Recent advances in AI, especially probabilistic genotyping, have significantly expanded the capacity to re-evaluate evidence previously deemed inconclusive. By explicitly modeling uncertainty and incorporating all available genetic data, these methods offer a more transparent and statistically grounded approach to forensic inference.

A DNA profile is expressed through numerical markers showing allele variations at specific genetic loci (20). When used in criminal cases, DNA evidence refers to biological traces at the crime scene, such as blood, saliva, semen, and skin cells, which are later compared with a suspect or other reference samples. If properly collected, stored, and analyzed, these profiles can link a person to a crime with very high accuracy or exclude them entirely. When evaluating DNA evidence, important differences exist between US and European standards governing its admissibility. The US has centered on how DNA profiles are collected and used within constitutional and statutory limits, while the European discussions are often focused on the retention and deletion of DNA profiles in state databases using the Marper standard (21). In the US, DNA profiles are analyzed through the lens of the constitutional protection against illegal search and seizures (Fourth Amendment of the Constitution), while in Europe, the focus is on the privacy protections in terms of the provisions of the European Convention of Human Rights (ECHR) (21).

## US experiences

In the US, the use of DNA evidence is regulated and applied separately at the federal and state level through the Rules of Evidence and the Innocence Protection Act of 2004, which allows for free post-conviction DNA testing to prove innocence. Furthermore, the US Constitution does not explicitly guarantee a general right to post-conviction DNA testing, and such access is primarily provided through statutory frameworks. These rules prescribe how a criminal procedure can be reopened and what admissibility standards are applicable in evaluating DNA evidence either in post-conviction or in primary proceedings. On the other hand, the novelties in easing the access to post-conviction testing, provided by the Innocence Protection Act (22), allow prisoners in state custody to seek supplementary DNA testing under defined conditions such as preserved DNA evidence with an intact chain of custody, and testing capable of producing material evidence that could show a reasonable probability of innocence. Furthermore, this Act requires preservation of biological evidence in federal cases and funding from the state, where prisoners cannot carry the costs for the supplementary testing of the DNA material.

To illustrate how DNA evidence is admitted in the US, the procedure allows for two admissibility standards: the Frye Standard from the case of *Frye v. the United States* and the Daubert Standard from the Supreme Court decision of *Daubert v. Merrell Dow Pharmaceuticals, Inc* (22,23). The former helps the court to assess the methodology used in the analysis of the DNA material and its acceptability in a particular scientific field (21). Under the Daubert standard, the judge acts as a gatekeeper and decides whether the expert's reasoning and methodology are scientifically reliable and properly connected to the facts of the case. In assessing forensic DNA evidence, the court considers whether the method can be tested, whether it has been peer reviewed, whether its error rate is known, whether clear operational standards exist, and whether the method is generally accepted within the relevant scientific community (23). Concerning modern DNA testing, the United States Supreme Court in the case of *DA Office v. Osborne* from 2009 recognized DNA testing as uniquely capable of both confirming guilt and correcting wrongful convictions, making it central to post-conviction review (24). However, in the post-conviction context, in which DNA testing was most prominent, the Supreme Court has recognized a tension between the finality of the judgment and proving of actual innocence of the perpetrator. In other words, it poses the question of whether there is enough evidence to convict the person, and how the court balances the conviction *vis-à-vis* the proposed evidence.

When DNA is used in innocence work, the issue is whether a convicted person can obtain access to biological material and request new testing after the trial has ended. In the US this is mainly regulated by federal and state post-conviction laws, while constitutional review remains limited. Such standards can be found in *Maryland v. King,* which shows that DNA is treated differently from biometric data, and is more sensitive than ordinary biometric data because it contains genetic information and raises stronger privacy concern (21,25).

## European experiences

The treatment of DNA evidence in the continental European jurisdictions is regulated both on the national and supranational levels. Supranational standards are promulgated by the EU, the CoE recommendations, as well as the jurisprudence of the European Court of Human Rights (ECtHR). The CoE Convention on Human Rights and Biomedicine (Oviedo Convention), which addresses genetic data rights and the individual consent, provides a special protection of genetic data, and defines them as a special kind of data, which require a special approach and treatment (26). However, the focus here will be on the shared CoE recommendations: R(87)15, R(92)1, and the Convention for the Protection of Individuals concerning the Automatic Processing of Individual Data, known as the ETS No. 108 and the update of the Convention, the new Convention 108+.

Recommendation Nm. (87) 15 from 1987 regulates the use of personal data in the police sector, including data-sharing, supervision, and international cooperation for law enforcement purposes (27). For DNA data, it requires that retention be limited to what is strictly necessary for preventing a concrete danger or investigating a specific criminal offense. It also stipulates that storage of such data be limited to only necessary data, as well as poses an obligation of deletion of any unnecessary collected data. The CoE Recommendation No. R(92)1 on the use of DNA analysis in criminal investigations adopted in 1992 emphasizes the necessity of clear and transparent laws of member-states governing the collection, storage, and utilization of DNA samples, and imposes quality control of all gathered DNA data (25,28). Convention 108+ modernizes the original data-protection framework by extending safeguards to both automated and manual processing of personal data. Genetic data may be processed only when the law provides appropriate safeguards, including independent supervision, limits on data transfer, and mechanisms for international cooperation (29).

On EU level, DNA analysis in criminal investigations is governed by a more complex framework, aimed at enabling cross-border information exchange while preserving data protection safeguards. Cross-border collaboration against terrorism, transnational crime, and illegal migration by allowing comparison of DNA profiles (with particular focus on the non-coding parts of DNA) is regulated by the Prum Convention (30). Furthermore, the EU Council Decision 2008/615/JHA enables access to national databases by preserving the distinction between access to DNA profiles and access to the personal data linked to those profiles (31). Further, the Law Enforcement Directive complements this framework by protecting fundamental rights when personal data are processed by criminal law enforcement authorities (32,33).

Concerning the unification of the national standards for the treatment of DNA evidence materials, most relevant is the jurisprudence of the ECtHR. The ECtHR addresses related forensic science issues through the lens of the right to privacy and the right to a fair trial, thereby unifying the standards for all jurisdictions. The major difference in the treatment of DNA evidence (and in this matter AI-assisted analyses) in Europe is that such evidence is not admitted through a Daubert/Fray-style admissibility test, as in the US Instead, judges assess credibility individually. Through its jurisprudence, the ECtHR has cemented that DNA has a specific status of personal data. However, the ECtHR has established three admissibility standards – the pronged test, called the Marper test, named after the *S. and Marper v. the United Kingdom* case, to take into account when assessing admissibility of forensic evidence. The first criterion is the legality of the measure, or whether the measure was envisaged in the national law. The second criterion is the proportionality of the retention period of the collected DNA evidence. The period of retention must be proportionate to the committed crime; severe crimes get a longer retention period. The third criterion is a periodic review and deletion of the DNA samples. National authorities must satisfy two basic requirements when processing DNA-related information: the interference must have a basis in domestic law, and law must be clear, foreseeable, and accessible. If these conditions are not met, the measure may violate Article 8 of the European Convention on Human Rights (34). In conclusion, European experiences focus more on genetic privacy, which implies that DNA materials may be used for law enforcement purposes, but only within a clear and transparent legal framework that is necessary, proportionate, and effectively supervised.

When it comes to AI-assisted analysis of forensic evidence, in Europe, such machine systems are already used in practice, especially probabilistic genotyping software for complex DNA mixtures. However, as opposed to the US, there is yet no well-developed body of European supranational case law specifically addressing it as such, nor any standards of admissibility. In Europe, the use of AI is focused more on investigative proceedings rather than on judicial proceedings. According to Fair Trials International, facial recognition is currently the most common AI-based tool used by security forces in Europe (35). However, not all surveillance systems rely on AI, but several European states have experimented with biometric and predictive tools in criminal investigations. Belgium, for example, tested automated facial-recognition surveillance at Brussels Airport, but this practice was later restricted by amendments to the Law on Police Services, which limited the creation of databases enabling comparison between camera images and existing biometric data (36). France has adopted a more permissive approach. Its Internal Security Code allows facial-recognition tools in border-control systems, while criminal-procedure rules permit automated processing of personal data in serious-crime investigations for detecting offenses, gathering evidence, identifying perpetrators, linking related events, and verifying identities through fingerprint databases (37-39).

## When algorithms “testify:” evidentiary and procedural implications

In traditional criminal trials, DNA evidence enters through expert testimony. AI can aid DNA analysis by enhancing the reasoning and the fact-finding process. However, the question here is whether the Frye and Daubert standards are applicable to AI-assisted forensic tools, and if they subject to Article 702 of the Federal Rule of Evidence. The short answer is yes; the tools and their derived results are subsumed under these standards and evaluated as any new scientific evidence or a scientific method. For the AI-assisted analysis to be used as evidence, it must satisfy the Daubert criteria on reliability, testability, error rates, and reliable application by the expert using the probabilistic genotyping software: STRmix and TrueAllele. Established judicial practice in the US indicates that courts have treated these systems the same as expert testimonies or any other forensic expertise. This means the admissibility question is whether the scientific method and its application are sufficiently reliable or generally accepted. According to the review of the National Institute for Standards and Technology, DNA mixture interpretation is scientifically complex, and probabilistic genotyping emerged as a response to the weaknesses of more subjective mixture interpretation (40).

The case of *United States v. Gissantaner* from 2021 demonstrates how the Daubert standard is applicable in practice (41). The case revolved around whether touch DNA evidence generated through the STRmix software was reliable enough to be admitted in a federal prosecution. The prosecution’s case relied heavily on a small amount of mixed DNA materials recovered from the weapon. A forensic expert asserted with high certainty that these fingerprints were likely to belong to the defendant, even though there were two other sets of fingerprints belonging to an unknown person. The court found that the prosecution tried to overstate the claims from the expertise and STRmix results as solid scientific proof. Further, it deemed that the DNA sample was a complex, low-template touch DNA mixture with at least three contributors, where, according to the results of the software, the defendant was treated as only a minor contributor at about 7% of the total DNA material. Testing and validation in this case were insufficient, and, for the current specific sample, only 49 picograms were attributable to the defendant, which is roughly 8-9 human cells, and there was no reliable testing or validation to justify admission in a criminal prosecution.

The case of *State v. Simmer* from 2019 is probably the first case where the state Supreme Court legitimized the result derived from probabilistic genotyping software such as TrueAllele to be sufficiently reliable to be admitted as evidence in court under the Daubert standard of admission of scientific evidence (43). The case revolved around a murder trial, where the prosecution relied heavily on DNA evidence recovered from a handle of a knife and door knob from the interior part of the domicile. Several DNA evidence traces were found, and the TrueAllelle software was used to ascertain which DNA fingerprint was most recovered. The key issue was whether this software can reliably link the defendant to the crime. The court admitted the evidence derived from the software, concluding that admissibility was granted based on the applied method, which was scientifically tested, validated, peer reviewed, and generally accepted in the scientific community. Evidently, this judicial decision marks an important step in the normalization of AI-assisted forensic interpretation, and a shift from the traditional human DNA interpretation of forensic evidence.

In the case of *People v. Wakefield*, the appeals court confirmed the conviction of murder and robbery in the first degree, deciding that there was no evidentiary error in the proceedings (44). The case is relevant from the aspect of accepting AI-assisted forensic expertise using the Frye standards (44). The central issue was whether DNA-mixture evidence generated by TrueAllele was admissible under the Frye standard. The court accepted the evidence, finding that the probabilistic genotyping method was generally accepted within the relevant scientific community. It also rejected the defense request for disclosure of the software source code, holding that admissibility depended on the acceptance of the method, not full access to the code. This case shows how courts may admit AI-assisted DNA evidence even when the underlying software remains partially opaque.

The case of *Lydell Grant v. State of Texas* is different from the cases presented above. It was not an appellate decision but a case of a granted new trial based on new evidence that led to exoneration. The case illustrates the transformative impact of AI-assisted DNA analysis in innocence litigation (45). It revolved around a first-degree murder, for which the defendant was convicted in 2012 and sentenced to life imprisonment, primarily on the basis of eyewitness identifications. Further, DNA recovered from beneath the victim’s fingernails produced a multi-contributor mixture that could not be interpreted using traditional methods and was therefore excluded from meaningful consideration at trial. Years later, reanalysis using an AI-driven probabilistic genotyping system conclusively excluded Grant as a contributor with an exclusion likelihood ratio on the order of one in ten trillion and inferred a reliable DNA profile of an unknown male contributor. The new evidence was enough to accept the claim of *habeas corpus* relief, which is a post-conviction legal remedy in US law. This profile ultimately led to the identification and confession of the true perpetrator, resulting in Grant’s exoneration after nearly a decade of wrongful incarceration. Evidently, probabilistic genotyping can serve as a corrective function in criminal justice by reassessing mixed DNA evidence, excluding innocent convicted persons and identifying the true perpetrator.

The American experiences are very forward-looking. The Frye and Daubert standards and admissibility tests allow for human and AI-assisted DNA analysis only if certain indicators are met. The demonstrated casework clearly shows that such genotyping software is considered new evidence and a new method, and this is admissible in court. Stemming from innocence work, it can be concluded that such software can immensely improve the chances of getting an acquittal by reassessing scientific evidence assessed by a human.

The European experiences do not indicate any cases with direct involvement of AI-assisted DNA analysis to be submitted as evidence. The case of *Glukhin v. Russia* revolved around a protester who staged a peaceful protest (46). The police used advanced facial recognition technology on captured CCTV footage to identify and arrest the person. The main concerns in this case were whether the use of facial recognition and biometric data to identify and arrest the applicant violated his freedom of expression. The ECtHR focused on the intrusive nature of facial-recognition technology and the absence of adequate safeguards in domestic law. It found violations of Articles 8 and 10 of the ECHR, holding that the use of this technology interfered with the applicant's privacy and freedom of expression. The Court also emphasized that the processed data revealed the applicant's participation in a peaceful protest and therefore indirectly disclosed his political opinion, which required heightened protection as sensitive personal data.

The case of *Ligue des droits humains* revolved around the lawfulness of large-scale collection, transfer, automated processing, and retention of passenger name record data for the purposes of combating terrorism and serious crime (47). The main concern of the Court of Justice of the European Union (CJEU) was that the checking relied partly on automated AI-surveillance of all passengers, including advanced assessment by comparison with databases and predetermined criteria, long retention periods, and possible extension to intra-EU travel. What is relevant here in connection to AI-assisted DNA analysis is that the judgment presents the CJEU’s position on automated processing of sensitive personal data in criminal justice. Further, it supports the argument that if AI is used in DNA interpretation, it must be strictly necessary, purpose-limited, non-discriminatory, humanly reviewable, and surrounded by safeguards against black-box decision-making, rather than treated as automatically acceptable simply because it is technologically advanced.

The case of *S. and Marper v. the United Kingdom* revolved around the police retention of DNA profiles of individuals arrested but not convicted. The Court condemned the UK’s blanket and indefinite retention of DNA data as disproportionate interference with privacy, especially for minors and persons who had not been convicted (34,48).

The case of *Gaughran v. United Kingdom* is another case inspired by the Marper case where the Court extended the logic of *S. and Marper* to Northern Ireland, and ruled that biometric-data retention rules had not been reformed. The Court found that the blanket and indefinite retention of DNA profiles was disproportionate because the law did not account for the seriousness of the offense or provide effective review mechanisms (34,49,51).

These cases showed that the Court and the developing standards insisted that the advanced technological forms, whether for the purposes of criminal investigation or identification, must be in line with the fundamental rights. Therefore, the use of AI-assisted software analyses should rest upon the principles of legality of foreseeability, have adequate safeguards, and be subject to a periodic review. US: experiences offer a more developed and litigation-oriented framework for the use of DNA evidence and AI-assisted DNA interpretation because admissibility of such evidence is analyzed through the Frye and Daubert standards, whether in primary or post-conviction proceedings. The analyzed cases indicate that the US judicial practice is evolving, and AI-assisted software analyses are permitted as scientific evidence if they are in line with the criteria set forth by both standards. As opposed to this, in Europe, there are no admissibility criteria of scientific evidence similar to these standards. The American model is more open to validating new forensic technologies, including probabilistic genotyping directly in the criminal procedure, whereas the European model is more cautious and rights-oriented, with more emphasis on legality than on admissibility.

## Current policy approaches to AI in the legal system

Policymakers increasingly recognize AI use in criminal justice. Emerging regulatory frameworks emphasize human oversight, transparency, explainability, and protection of fundamental rights. Importantly, current policies reject fully autonomous decision-making (ADM) in criminal justice and stress that AI must only assist and not replace humans as central decision makers (35). In forensic DNA analysis, this principle translates into mandatory expert oversight and legal accountability. In Europe, AI and ADM systems are used to assist, influence, or inform law enforcement and criminal justice decisions according to the report of Fair Trial International (35). This trend has been growing over the last decade due to the increasing availability of data and more advanced data analytics tools. AI and ADM systems used by law enforcement and in criminal justice can have a very serious and real impact on people’s lives. In the Netherlands, ProKid software is used for predictive policing, ie, risk assessment of criminality of young adults and children. However, research has indicated that ProKid software does not actually predict the likelihood of criminality, but the likelihood of a child being registered on a police system in relation to a crime. The ProKid algorithm was developed using police records concerning 31 769 children aged 12 to 18 who had been registered in 2007 as suspects, victims, or witnesses (54). The software generated risk assessments by taking data from two police databases: one containing criminal records and evidence, and another containing contextual information, metadata, and observations recorded by criminal-justice actors (35,55).

In Italy, the Dynamic Evolving Learning Integrated Algorithm, or Delia, was until 2021 used by the police for geographic and individual crime prediction by analyzing criminal behavior (35). The software created and compared profiles from millions of combinations of variables in order to link crimes committed by the same individual or a criminal gang, as well as assisted in the interpretation on target types (56). The system processes identifying and contextual details, including age, physical appearance, clothing, accent, possible weapons, and vehicle information (type, model, make, and license plate). Furthermore, the software uses ethnicity data and is used in AI-assistive tools in prosecutions (35). However, this software was short-lived, and during the COVID-19 pandemic, the Department of Public Security of Italy’s Interior Ministry hinted that it aims to develop a national predictive policing system based on KeyCrime's experience. By 2023, the Central Anticrime Directorate of Italian National Police stated that the Giove project was not yet operational and still in the “feasibility study” phase, according to information provided after a transparency request was filed (57).

Further, the EU AI Act aims to make AI systems within the EU safe, lawful, and in conformity with fundamental rights (58). Its relevance to AI-assisted DNA analysis is that it envisages for transparency, human oversight, risk management, data governance, and accountability when any AI tools are used in criminal-justice settings. AI-assisted DNA software, such as TrueAllele or others, cannot be treated as a black box, meaning that there must be validation, oversight, and the possibility of challenge. The final version of this act, promulgated in 2024, includes the establishment of the European Commission’s AI Office, which will be responsible for monitoring the implementation of the AI Act.

The CoE Framework Convention on Artificial Intelligence and Human Rights, enacted in 2024, complements the EU AI Act, and further imposes binding human rights obligations for organizations and individuals for ethical and transparent use of AI (59). Same as the AI Act, this conviction reiterates the glass box usage of AI algorithms. It envisages transparency of AI uses and human oversight mechanisms, as well as accountability for data controllers (59). Furthermore, the Conviction prohibits these AI systems from performing any kind of discrimination or producing inequality, and envisages special protections for personal data to prevent misuse. Important for AI-assisted DNA analysis, although not directly mentioned, is that in this Conviction, all member states ensure reliability and promote innovation aligned with human rights protections envisaged in the ECHR, regarding Articles 10, 11, and 12 (59).

In the United States, there is no comprehensive regulation of AI such as the EU AI Act, only judicial precedents. The closest legal document is the White House AI Action Plan, promulgated in 2025, which includes criminal justice-related AI use, under Combat Synthetic Media in the Legal System (60). The Action Plan stipulates that AI-generated media can create challenges for courts and law enforcement in terms of fake evidence. Therefore, these actors should have the proper tools to address these AI-related risks. It recommends that NIST consider turning its deepfake evaluation work into a formal guideline and voluntary forensic benchmark, and that the Department of Justice (DoJ) should issue guidance to agencies involved in adjudications and comment on possible deepfake-related additions to the Federal Rules of Evidence. Furthermore, the M-25-21 OMB Memorandum for Federal Agencies is an internal federal government document for the use of AI within the agencies. This document specifies in Section 6 that AI should be used for identification of criminal suspects, forecasting of a crime, application of biometric identification, facial reconstruction based on genetic information, and more (61).

The DoJ Artificial Intelligence and Criminal Justice Final Report recognizes that AI may improve the efficiency and consistency of criminal prosecution and other sentencing-related functions such as bail and pretrial decisions, probation, predictive policing, and forensic analysis (62). However, the report warns that the overreliance on such tools can create inequalities and, in some cases, wrongful prosecution. This can further undermine transparency, and threaten privacy, civil rights, and civil liberties if used without strict safeguards. The report cautions that AI systems trained on criminal justice data have the capacity to reproduce existing inequalities. For example, if a risk-assessment tool uses housing stability or prior arrests as indicators, it may indirectly reflect biases from housing markets or policing practices. As a result, such systems may reinforce, rather than correct, structural disparities (62).

Although different, the US and the European approach are forward-looking and innovative. The European standards build a comprehensive and binding framework centered around the fundamental human rights envisaged in the ECHR. The use of AI in predicting criminal behavior or determining sentences is forbidden, and AI tools used by law enforcement or courts are classified as high-risk category, requiring human oversight, and mandate transparency about error rates and system assumptions so that affected people can contest decisions. On the other hand, the US approach is more practical and non-statutory, but built through judicial practice. Federal policies encourage agencies to enhance and normalize AI uses, as well as perform impact assessments and involve human reviewers. However, there is no unified regulatory legislation such as the EU AI Act.

## Documented AI use in post-conviction review and investigations

AI-driven forensic tools have also been applied in post-conviction review. Policy evaluations in the US have documented cases where probabilistic genotyping altered the interpretation of evidence used in prior convictions, revealing that results previously presented as strong inculpatory evidence were substantially weaker. When it comes to post-conviction work and the ability of AI to influence or speed the process up, the only avenue that should be explored is the reopening of the criminal procedure. The application of AI in this area, as suggested in the standards above, can be viewed from the perspective of accepting a new scientific method as new evidence that is strong enough to reopen the procedure. Consequently, this aspect shaped important judicial developments on post-conviction reopening, particularly in defining when new scientific methods can justify revisiting a final conviction.

Even today, there is no academic or hermeneutical consensus as to what new scientific evidence is and how it should be evaluated (63). The classical criminal law recognizes three types of new evidence: *noviter productae* (pre-existing evidence, never evaluated or presented at the trial), *noviter repertae* (evidence that has emerged after the finality of the judgment), and *noviter cognitae* (elements collected during the trial but not yet assessed). However, Luparia argues that in judicial practice only *noviter repertae* evidence is favored by the courts when motions for reopening of the procedure are being filed (63). Pituritti and Luparia explain this restrictive approach by favoring traditional preference just for the sake of the stability of final judgments and the preservation of legal certainty in criminal justice (63). Many jurisdictions have undergone changes on the correct assumption that as technology changes, there are many new and modern ways to retest already presented evidence, and that should constitute a *novum*.

In Europe, proving innocence by reopening the procedure can be filed on many grounds (false testimony, false expertise, official misconduct by the actors of the procedure, new evidence, trial *in absentia,* and after a judgment of the ECtHR or the Constitutional Court, where some infringement of a procedural right is detected) (2). New scientific evidence relies on newly developed and highly specialized scientific methods, which are gradually changing and evolving. Gathering new evidence is one side of the coin, and how it is defined in the law is another. However, a breakthrough was made by the Italian Court of Cassation, in the landmark *Ghiro* case (64). The court developed criteria for assessing new scientific methods. The court accepted that the new scientific method can serve as a *novum* as it did not exist during the trial. In the *Ghiro* judgment, the Court of Cassation stipulated that the method must be genuinely new, scientifically reliable, accepted, and published within the relevant scientific community, applicable to evidence already examined at trial, capable of producing new results, and sufficiently significant to support a different outcome (65).

In Croatia, there are no unified judicial directions on how to assess new scientific methods or evidence. However, the County Court in Split accepted a new method as new evidence upon which a reopening of the procedure can be granted. The applicant had been convicted of document forgery and sentenced to two years’ imprisonment. In the reopening request, he relied on a new graphological report prepared with a method that had not been available during the original trial. The expert report concluded that the signature on the vehicle purchase agreement almost certainly did not belong to the applicant. Since no graphological expertise had been conducted during the first trial, the court considered this new finding capable of supporting an acquittal (66). This approach is in positive direction, and is in line with the Italian standards established in the *Ghiro* case. Although *Ghiro* evaluated newly presented scientific evidence, and established the admissibility criteria for forensic DNA evidence, such practice was not detected during the analysis of the judicial practice. No unified position exists on how to evaluate new evidence, especially scientific evidence, using the Ghiro method.

Describing the US experiences, Garret and Rudin state that although in several cases, the court has accepted AI-assisted analysis as evidence by applying the Daubert standard, it is deeply problematic when the courts are applying the black box model (10). Although they deem probabilistic genotyping as a very promising new method, it raises serious fair trial issues if criminal justice actors cannot examine the source code and the reasoning behind the conclusion reached by AI-assisted software (10). They champion more transparent and ethical safeguards of AI use, especially in criminal cases, where the prosecution should have to justify any use of AI, because interpretability is essential for protecting the credibility of the procedure. They encourage the implementation of the glass box model because such a model is aligned with the principles of discovery of evidentiary material and allows the parties to cross-examine or cross-analyze the algorithm used (10). The DoJ accepts that probabilistic genotyping is a new and powerful method, which, if properly used, can yield very precise results and must be operated through a controlled framework. Such genotyping is only a tool to assist the examiner, reporting a probability ratio not an absolute claim. In no way, it can replace human review or the human evaluation of the output (67).

The case of *People v. Collins* is a relevant example of using the AI black-box model, as the prosecution used a probability calculation to argue that the defendants must be guilty. The court rejected such claims because the numbers lacked a proper evidentiary foundation and risked misleading the jury with a false aura of scientific certainty (69). The court was concerned that the software code and ways of decision-making were not available to the public or to the defense. Although the court acknowledged the state’s interest in protecting expensive proprietary software, it emphasized that such opacity prevents defense experts from independently testing the prosecution’s theory (10,69). The software had been developed by New York City’s Office of Chief Medical Examiner, but the government refused to disclose its underlying code to the defense (10).

European jurisdictions need to adopt Frye/Daubert standards for evaluation of new scientific methods or evidence, even when such evidence derives from AI-assisted DNA analysis. Such standards would be indispensable for innocence-related work and will provide a filter for deciding when a new method can be considered scientifically reliable. This is precisely why such standards are compatible with the glass-box model of AI. In post-conviction review, such a model accepts the technological novelty and can be scrutinized by the parties and independently validated. In cases involving probabilistic genotyping, interpretability is indispensable if such evidence is to serve justice.

## Ethical and institutional accountability

Having analyzed US and European legal standards and jurisprudence, it can be concluded that as AI is becoming increasingly present in forensic genomics, ethical and institutional accountability must be treated as a legal requirement and not merely as a good practice. As seen through analyzed jurisprudence, justice actors are not scientists. This is why the most important part of criminal proceedings is the expert report. This report conveys very useful information to the parties of the procedure, and under the fair trial rules, these methods, reports, and findings must be disclosed to the other side (principle of discovery) to also include information favorable to the defendant, and be objective (71).

The CoE AI Conviction and the EU AI Act have adopted the glass box model, and require that all parties to the Conviction ensure transparency and oversight from any adverse impact of AI systems, especially if used for high-risk criminal justice purposes (11). Forensic companies and criminal justice actors working in the field of criminal justice must satisfy the high requirement of risk management, data governance, oversight, and cybersecurity. Here, the robust European legal protection is evident in choosing the glass box model and emphasizing transparency, oversight, and accountability (58). Translated into innocence work or in post-conviction settings, in any AI-assisted forensic analysis, the developer might bear responsibility for defective design or any misuse. In Europe, the protection is visible, but it is unclear how AI-assisted analysis is treated in the criminal procedure: as new evidence, a new method, or both? In the US, the use of both models is permissible, so the system can be called hybrid. However, as opposed to Europe, the US lacks a robust legislative framework to introduce safeguards of AI use for criminal justice purposes.

Also applicable here is the biomedical ethics principle of minimizing harm, which is developed in medicine with the notion that the welfare of the human being must prevail over the interests of science or institutional efficiency, as reflected in the Oviedo Convention (26). AI-assisted analyses can improve with great certainty and speed, but the more enhanced analytical capacity cannot justify their use unless the system is very reliable and safe. This is further stipulated in the UNESCO Conviction on Ethics of AI, which emphasizes accountability and human oversight over opaque authority (67).

When it comes to the application of the fair trial principles, Strasbourg jurisprudence is very much applicable through the principles of adversarial procedure, (non)disclosure of evidence, protection of privacy, and treatment of DNA as special data. The jurisprudence of AI usage, naturally, has yet to be developed. In the US, internal DoJ memorandums indicate that ethical safeguards, transparency, and human responsibility are needed to safeguard the integrity of evidence in criminal prosecution (67). More concretely, it is stated that probabilistic genotyping is only a tool that can assist the DNA analyst, and cannot replace human review of the output of the reporting. Furthermore, the guidelines forbid ethical overreach. This refers to the statement of the prosecution that DNA evidence has zero error or that the likelihood ratio proves absolute identification or source attribution. It also urges prosecutors not to use expressions such as reasonable scientific certainty (67). Such guidelines introduce epistemic humility, especially in probabilistic genotyping. Garrett explains that AI tools used in criminal trials should be subject to a strong requirement of interpretability and grounded in existing constitutional fair-trial protections and reinforced through legislation or regulation (10). He further urges US legislators to follow the EU approach and label AI usage in criminal justice as high-risk.

Although in many cases in the US the judges opted to admit the black box model, the presumption against it should be strong, and the prosecutor should be able to explain the process of how such results were deducted. The glass box is necessary from the aspect of safeguarding the rights of defendants, and they should have the right to cross-examine the expert evidence against them. After all, the burden of proof is always on the prosecutor, which should be able to justify the use of AI systems.

## Conclusion

The title of this article was chosen to demonstrate to a wider audience that AI-assisted DNA analysis is emerging from a forensic reality and has a clear potential to reform criminal proceedings. We showed how AI can be used in assistive capacity and highlighted possible implications that can arise in the criminal justice systems and jurisprudence. Probabilistic genotyping, as the most used AI-assisted method, has most certainly improved the interpretation of complex DNA samples, supplementing the traditional human-led methods. It more accurately determines the DNA proportion that can be attributed to the defendant and provides information on possible traces of third-party DNA on the material.

When it comes to admitting such evidence, the central legal issue is which conditions can be applied to evaluate its use in court and how to apply them. The US approach is quite practical as it has developed two applicable standards of scientific evidence: the Frye and Daubert standards. These standards measure the reliability of the methodology, the known error rates, peer acceptance, and expert application. They enable a thorough judicial filter, and seen through US jurisprudence, such AI-assisted evidence has been accepted for trial and jury evaluation. Europe lacks such unified standards, either on the supranational or national level. Instead, the treatment of scientific evidence is supranationally considered and rendered through the position of the ECtHR on DNA materials as a special type of evidence, and their ramification, storage, and collection. This approach is human-rights centered, and pertains more to the deletion and storage of DNA data rather than admittance. The European legislation consists mainly of supranational documents that classify the areas where AI use is considered low-risk or high-risk, and criminal justice is a high-risk area for human rights. This is acceptable because the European policymakers prefer the glass-box AI software, meaning that every AI analysis and results must be explained fully in court. In national jurisdictions, however, the *Ghiro* case in Italy and the emerging practice in Croatia, indicate that the admissibility models are slowly shifting, and the Frye/Daubert standards are slowly being implemented in national jurisdictions to assess such evidence, including the AI evidence. Therefore, on a supranational level, European jurisdictions should adopt standards for evaluation of scientific evidence similar to the Frye and Daubert standards in order to ease the admissibility of scientific evidence and AI-assisted-derived analyses into criminal proceedings.

The software used in AI-assisted analyses only provides a probabilistic ratio rather than certain results, and this is the acceptable standard in both the US and Europe. However, there are fair trial concerns about the transparency of the AI deduction process. Therefore, the use of AI software must remain transparent and open to adversarial testing. The glass-box approach should be the right model to safeguard constitutional guarantees.

However, the European approach to AI is normatively superior, with the enactment of the EU AI Act and CoE Convention, but procedurally incomplete, as it lacks admittance standards. The EU AI Act treats any use of AI software in the area of criminal justice as high-risk, and adopts the glass box method, which requires transparency, oversight, and human accountability. On the other hand, US experiences offer a stronger procedural pathway through the patchwork of jurisprudence and internal federal memorandums, rather than comprehensive laws. The US still lacks a federal binding AI Act to provide guidance and ethics in the use of such systems. However, until that, the US is relying on case law and judicial development.

The available resources show that AI-assisted DNA analysis can improve the interpretation of complex biological evidence and strengthen post-conviction review only if its use is embedded within transparent, scientifically validated, explainable, and procedurally accountable legal frameworks that preserve due process and equality of arms. The results provided by such systems should be a subject of thorough scrutiny and transparency and supported by rigorous scientific explanation and validation.

In innocence work and post-conviction review, the most important element is new evidence or new facts, which have a decisive power to reopen the criminal proceedings. In current judicial interpretation, only DNA evidence has such power. Therefore, retesting previously examined biological evidence with scientifically superior methods, which can constitute as new evidence, may lead to the creation of a general *novum* or new evidence, which is capable of overturning a wrongful conviction. As a key conclusion, any reform should aim at technological enthusiasm and fair-trial rights, privacy, and protection against wrongful convictions.
